# High Altitude Population Neonatal and Maternal Phenotypes Associated with Birth Weight Protection

**DOI:** 10.1038/s41390-021-01593-5

**Published:** 2021-06-08

**Authors:** Padma Dolma, PT Angchuk, Vandana Jain, Vatsla Dadhwal, Dalvir Kular, David J Williams, Hugh E Montgomery, Sara L Hillman

**Affiliations:** 1 Sonam Norboo Memorial Hospital, Leh, India; 2 All India Institute of Medical Sciences (AIIMS), Delhi, India; 3 University Hospital Lewisham NHS Trust, United Kingdom; 4 University College London Institute for Women’s Health, London, United Kingdom; 5 Centre for Human Health and Performance, University College London, United Kingdom

## Abstract

**Background:**

States which reduce fetal oxygen delivery are associated with impaired intrauterine growth. Hypoxia results when barometric pressure falls with ascent to altitude, and with it the partial pressure of inspired oxygen (‘hypobaric hypoxia’). Birth weight is reduced when native lowlanders gestate at such high altitude (HA)- an effect mitigated in native (millennia) high altitude populations. Studying HA populations offer a route to explore the mechanisms by which hypoxia impacts fetal growth.

**Methods:**

Between February 2017 and January 2019, we prospectively studied 316 pregnant women, in Leh, Ladakh (altitude 3,524m, where oxygen partial pressure is reduced by 1/3) and 101 pregnant women living in Delhi (low altitude, 216m above sea level).

**Results:**

Of Ladakhi HA newborns, 14% were small for gestational age (<10^th^ birth weight centile) vs 19% of newborn at low altitude. At HA, increased maternal body mass index, age and uterine artery diameter were positively associated with growth >10^th^ weight centile.

**Conclusion:**

This study showed that Ladakhi offspring birth weight is relatively spared from the expected adverse HA effects. Furthermore, maternal body composition and greater uterine artery size may be physiological HA adaptations and warrant further study as they offer potential mechanisms to overcome hypoxia related growth issues.

## Introduction

Birth weight is an important determinant of fetal and infant survival (1–4). Small newborns are particularly vulnerable to morbidity and mortality (5–7). Conditions which reduce fetal oxygen delivery (such as significant maternal cardiac disease) are associated with lower birth weight (8). Exploring the pathogenesis of this impact in disease states is difficult. One approach is to study otherwise healthy individuals exposed to the environmental hypoxia which results from residency at high altitude (‘hypobaric hypoxia’), amongst whom birth weight is reduced by 100g per 1000m ascent beyond 1500m (9–11). Such impacts are seen especially amongst native lowlanders. Native highland populations (historically exposed to hypobaric hypoxia for millennia) have undergone selection for advantageous genetic variants (12), and this might contribute to the partial mitigation of HH impacts on fetal growth seen in such populations such as those in the Andes (13). Likewise, babies born to Tibetan women are < 300g and <500g heavier than those born to Han Chinese (native lowlander) women at 2,700m and >3000m respectively (14).

In this regard, a variety of advantageous phenotypes may have been selected for. The uterine artery (UtA) supplying blood to the growing pregnant womb increases in diameter as gestation advances (15). At 20 weeks gestation at altitude, UtA blood flow rises more in Andean than European native lowlanders (16), whilst 3^rd^ trimester UtA flow at high altitude (HA) is greater in Tibetans than Han Chinese(17). Increased UtA diameter and fetal birth weight in Andean women appears influenced by selection on the PRKAA1 gene, which encodes AMPK, a central regulator of cellular energy metabolism (18).

Fetal genotype influences birth weight at low altitude, independent of maternal genotype (19). However, selection pressure on such elements at HA remain little studied.

The Ladakhi people of the Indian Jammu and Kashmir regions represent an ancestral HA population, dwelling between the Karakoram and Himalayan mountain ranges at ≥3400m. They share some elements of ancestral history with neighbouring Tibetans (20), but have been less extensively studied. The few papers investigating birth weights from this population are more than 15 years old (21, 22) and, whilst supporting the protective influence of Tibetan ancestry on birth weight, they failed to confirm the same protective effect on Ladakhi offspring. Wiley et al reported mean birth weight of 168 Ladakhi babies to be 2764g, but gestational age was not measured. Instead, fundal height and head circumference were used as proxies (21).

We thus set out to perform a larger prospective observational study in order to elucidate the effects of HA hypoxia on birth weight in a single native HA Ladakhi population. Specifically, we sought to:

i) Report the association of maternal and offspring anthropomorphic characteristics with birth weight impacts, and specifically that of increased UtA diameter
ii) Collect biological samples to investigate genetic and metabolic mechanisms that might help explain the phenotypes identified

## Methods

Two linked prospective observational cohort studies were carried out. Pregnant women were recruited from Sonam Norboo Memorial (SNM) Hospital, Leh, Ladakh (the HA site, 3,524 metres) from the antenatal clinics and antenatal ultrasound departments over a 2-year period (Feb 2017-Jan 2019). Infants born at the All India Institute for Medical Sciences, New Delhi (216m above sea level) were studied in parallel over a 1-year period (Jan 2018-December 2018). Ethical permissions were granted for the study from the Indian Health Ministry’s Screening Committee (HMSC) on the 7^th^ September 2016, the Office of the Chief Medical Officer Leh (3^rd^ August 2016), the All India Institute for Medical Sciences and the University College London research ethics committee (3634/002).

Pregnant women were eligible if they were aged over 18; not known to be genetically related to the father (First cousin or closer); having a singleton pregnancy; planning to deliver at the hospital; < 28 weeks gestation using last menstrual period (LMP) and in whom an estimated due date (EDD) could be confirmed by ultrasound. Where LMP was uncertain or where there was a difference of more than 5 days by an appropriate early ultrasound, the ultrasound determined EDD was used. Excluded were those pregnancies with obvious fetal structural or chromosomal abnormalities.

Pregnant women completed a questionnaire documenting their dietary patterns (meat eater/ vegetarian); and family, obstetric and medical histories (including smoking, alcohol, chronic medical problems and medications). Geographical ancestry was recorded with, where possible, birth locations for more than five generations. Blood pressure and anthropomorphic measures (height and weight pre-pregnancy and at attendance) were recorded using standard anthropometric techniques at the enrolment, booking appointment. UtA diameters were measured in longitudinal section at its crossover with the external iliac artery using transcutaneous ultrasound, between 18-24 weeks gestation (17) by a single trained operator at each site (4-MHz curved linear array probe ALOKA Prosound, Hitachi Aloka. Medical Ltd, Chennai, India) in Leh and a Voluson™ E6 (GE Healthcare, Chicago, Illinois) in Delhi. The process was repeated bilaterally, and values of six readings (three per side) recorded. Colour imaging was not used, so as to avoid artefactual increases in UtA diameter. A second operator verified 10% of images and calculated average UtA diameters.

Information concerning the birth process (mode of delivery) and the neonatal characteristics (sex, weight, head circumference, crown-heel length and APGAR score) were recorded. Arterial transdermal oxygen saturation was recorded by pulse oximeter within first 15 minutes of birth from both a finger and toe with babies lying, resting and not crying and in the absence of supplemental oxygen delivery.

Deliveries were classified preterm if <37 weeks gestation. All deliveries up to 42 weeks’ gestation were included in the analysis. Small for gestational age (SGA) infants were defined by a birth weight of <10^th^ centile according to the Intergrowth software package (https://intergrowth21.tghn.org).

## Statistical Analysis

Outcome measures were defined by cases- pregnancies affected by SGA versus controls (pregnancies with appropriately grown for gestational age (AGA) offspring). Analysis was first undertaken to compare maternal characteristics between SGA and AGA pregnancies in the HA and LA locations. Comparisons between HA and LA locations were also undertaken for the entire cohorts and then stratified by SGA and AGA outcome.

Statistical analysis was performed using the STATA 16 package. Normality was confirmed through visual representation of birth weight distribution by histogram plot. Maternal baseline characteristics are reported as means [standard deviation SD] unless otherwise stated. Unpaired t-test were performed and reported for baseline characteristics between women who delivered AGA babies and those that delivered SGA babies.

Birth weight centiles and birth weight centile ratios were calculated using Intergrowth standards as previously documented. Ponderal index was calculated as infant birth weight (g)*100/ birth length (cm)^3^. Logistic regression was used to identify factors contributing to the probability of SGA delivery in the HA population. Logistic regression results are reported as odds ratios with 95% confidence limits. Explanatory variables are assessed as significant at the 5% level. Univariable logistical analysis of maternal variables was first undertaken from the entire HA cohort. Maternal variables that retained significance in the first model were included in the final multivariable model to identify the association of UtA diameter and being an SGA case. Data were initially analysed by univariable logistic regression and those with the lowest P value used to generate the final multivariable model. Sensitivity analysis using forward stepwise regression confirmed the validity of this approach.

### Sample size calculation

Sample size was calculated prior to commencement of the study using the STATA statistical software package. At 90% power with a 0.05 significance level, 184 subjects would allow for detection of a 300g birth weight difference between SGA and AGA cases. This assumes a SD of 540g, which is based on previous studies (21, 22) with a 3:1 AGA: SGA allocation (so recruitment of 46 SGA cases). A planned sample size of 300 HA residents was thus selected based on a predicted SGA case rate of 15% SGA pregnancies in Leh (supported by earlier audit data) equating to 45 SGA babies. For the logistic regression model, 10 events (SGA outcomes) are required for each coefficient estimated, so 45 cases would allow for analysis of up to 4 coefficients in a final model.

## Results

Overall, 316 pregnant women were recruited in Leh (HA), and 101 in Delhi (LA) (1 year due to later start of enrolment). Over the two-year period, all women where gestational age could be ascertained and thought to be <28 weeks, were approached to be in the study. Maternal characteristics are presented in Table 1 and birth weight traits in Table 2.

In Leh, more than 96% of women considered themselves of Ladakhi descent (with a 5 generation or more documented history of living at altitude), whilst 10 (3.2%) described themselves as Tibetan. Birth weight was greatest in the Tibetan ancestry group (3.62kg) vs Ladakhi (3.14kg), p 0.0009.

Of AIIMS participants, the majority of women had lived in Delhi for more than 5 years, but more than half came originally from outside of Delhi area. Overall, 94/101 (93.1%) described themselves as Indoaryan ethnicity, 2 as Indo-European and 5 as Dravidian.

Characteristics of HA women who delivered SGA verses AGA babies were similar. Maternal ethnicity, parity, smoking status (no subjects disclosed being a smoker at HA), chronic illness and socio-economic (as evidenced through maternal education, occupation, income and educational status) did not differ (Table 1). No significant maternal illness was reported in either group (hypothyroidism was reported in both groups). At HA, maternal BMI (AGA 24.1 vs SGA 22.3kg/m^2^, p=0.002) and age (AGA 27.2 vs SGA 26.5 years, p=0.03) differed. Compared to women who delivered an AGA baby, those who delivered an SGA baby were statistically older and lighter.

Baseline maternal characteristics of women who delivered at LA revealed a similar pattern. Ethnicity, parity, age and smoking (one woman with an SGA pregnancy reported current smoking) were not found to be different between groups. Maternal BMI was different between groups (AGA 23.4 vs SGA 21.1kg/m^2^ [p=0.03]). Hypothyroidism was reported once in each group with one report of maternal hypertension in the SGA group.

Of the 316 HA women, UtA data were obtained in 221 (69.9%). Missing UtA data was due to the sonologist not being available to scan (n=29), women being outside of 18-24-week gestation period (n=34) or if the blinded analysis disagreed (n=32). Data were not used where the second operator disagreed on blinded analysis by >0.1cm.

At HA, mean UtA diameter was 56.5 [10.7] mm in AGA pregnancies vs 51.5 [9.3] mm in SGA pregnancies (p=0.009). Birth weights in the 187 AGA neonates where UtA data was available were similar to that in the overall cohort recruited (3.23kg). SGA pregnancies with UtA data reported (n=34) were also found to have similar birth weight (2.51kg) to overall study population (2.52kg in total SGA cohort) and SGA was reported at a similar rate (15%). In the SGA group there was no statistical difference in birth weight between those with UtA measurements (2.51kg) and those without (2.54kg), p= 0.69.

Univariable logistical regression analysis of maternal characteristics between AGA and SGA babies at HA revealed body mass index (BMI) and age to be significantly different between groups. Therefore, a final multivariable logistical regression model was run incorporating maternal BMI, age and UtA diameter in babies born AGA versus SGA at HA. Maternal UtA diameter remained statistically different between women who had an SGA baby versus those who had an AGA baby (OR 1.06 p=0.0001) with increased maternal UtA positively associated with having an AGA baby. (Table 3)

UtA diameter was statistically smaller in the LA group vs HA groups 47.3 [4.65] vs 55.5 [11.2] mm respectively, p=0.0001). In LA babies, UtA diameter was appropriately recorded in 61 (60.3%). In this group the average birth weight was 2.99kg in AGA pregnancies (n=47) and 2.43kg (n=14) in SGA pregnancies. There was no difference in size in the UtA diameter between AGA and SGA pregnancies (47.2 [4.9] vs 47.2 [4] mm, p= 0.97) in the LA cohort.

Neonatal data for HA and LA sites are presented in Table 2. At HA, the mean birthweight was 3.15 [0.44] kg, and 44 infants (14%) were classified as SGA (mean birth weight 2.52 [0.26] kg). SGA babies were significantly lighter than AGA babies 3.25 [0.39], p=0.00001. Mean gestational age (days) did not differ between AGA and SGA babies (275.3 vs 277.4 days respectively, p= 0.21). Mean birth length at HA was 49.9 [2.6] cm with SGA babies being significantly shorter 47.5 [2.4] cm vs AGA babies 50.3 [0.15] cm, p= 0.0001.

The mean birth weight in the LA cohort was 2.89 [0.4] kg, significantly less than the HA cohort (p=0.0001) with 19 (19%) infants identified as SGA (mean 2.43 [0.22] kg). Birth length was 48.1 [1.7] cm and SGA babies were again shorter at 47.1 [1.44] vs AGA babies 48.3 [0.19] cm, p= 0.0067. Babies were born earlier than HA counterparts (268.7 [8.7] vs 275.6 [10.4] days, p=0.0001). No significant difference was noted between gestational age that AGA vs SGA babies born (268.8 [8.9] vs SGA 269 [8] days, p=0.91).

Birthweight was greater in male vs female babies at both locations. Males at HA were heavier. than females weighing 3.21 [0.45] kg vs females at 3.1 [0.45] kg, p= 0.03. At LA, male vs female weight difference was not found to be statistically significant (p=0.19). Male/female numbers were similar at both HA and LA sites (HA male sex 45% vs LA 46%). Overall babies born at HA of Ladakhi descent were heavier (3.15kg vs 2.89kg, p =0.0001 and longer (49.9 vs 48.1mm p=0.0001) than their LA counterparts. Comparing just SGA pregnancies at HA and LA, significant differences between birth weights and length were not maintained.

At birth there was no statistically significant difference between baby APGAR scores at 1 minute between HA 8.26 [1.17] vs LA 8.47 [1.45], p=0.11 (Table 2). At HA APGARs at 1minute for SGA babies were 8 [1.26] vs AGA babies 8.3 [1.15], p= 0.21). SaO_2_ shortly after birth was lower at HA than LA (88.9 [8.32] % vs 92.5 [13.2] % respectively), p= 0.005. SaO_2_ did not differ between SGA and AGA babies at HA (88.7%), but initial SaO_2_ was higher in AGA than SGA babies born at LA (93.1 [13] % vs 88.7 [8.49] %, p=0.39) respectively, although not reaching significance.

## Discussion

We performed a comprehensive prospective study of a HA native population in relation to reproductive outcomes and compared this with a LA non-native population.

Individuals gestated and born at HA had a significantly higher birth weight than expected for an Indian population. The only other published study specifically of birth weight in the Ladakhi population reported significantly lower birth weights than those recorded in this study as well as shorter birth lengths with a much higher rate of low birth weight <2500g (27% versus 7.8%) (21). It is difficult to know the exact reason for this disparity. The previous study is >25 years old and tourism in the region has had a significant impact during this time, potentially altering parental diet and lifestyle. The sample size in our study is twice as large and was performed over a much longer time frame and may better represent the population especially given that almost all eligible pregnant women consented to be in the study.

Birth weights in Ladakhi subjects were on average 3.15kg, up to a maximum birthweight of 4.4kg. Within this population those individuals who identified as Tibetan still showed the greatest adaptation with an average birth weight of 3.62 kg. These figures correspond to other adapted high-altitude populations. A retrospective review of a cohort of South American birth weights revealed an average birthweight for high altitude Andean (longest adapted) subjects of 3.15kg (n=728), compared with their European (shortest) counterparts at high altitude at 2.96kg (n=167) (9).

Our data also support the idea that rates of poor fetal growth (as defined by Intergrowth reference standards) were lower than expected in the Ladakh population with significantly lower rates than documented by the recent Indian National family health survey 4 2015-16 (https://dhsprogram.com/pubs/pdf/FR339/FR339.pdf) for term low birthweight (LBW) individuals of 16.6% (versus 7.8% in our Leh population). The proportion of babies defined as small for gestational age (according to birth weight centile) at HA (14%) was lower than at LA (19%). Birth weight centiles take into account gestational age and this finding suggests adaptation in HA cohort is not purely a result of gestational age differences.

The differences seen in adaptation are driven by the appropriately grown individuals at high altitude. These infants are both heavier and longer than their counterparts born at low altitude, but this growth sparring effect is lost in infants born smaller than their expected weight with their birth length and ponderal index being equivalent whether at high or low altitude. This suggests that intrinsic mechanisms that influence body size and composition may have a role to play in positive adaptation at high altitude.

From this study, increased maternal body mass index and decreased age associated with birthweight offspring at HA. This is consistent with recognised maternal traits that associate with appropriate birth weight (23) but other classically recognised risk factors for low birth weight do not appear to be significant in this HA cohort (24), which is of interest given the concurrence with the neonatal anthropomorphic findings described above. The absence of retained significance in the multivariable model along of other classic risk factors for low birth weight in this high altitude population is in keeping with earlier high altitude work from Colorado that associated high altitude as an independent risk factor influencing birth weight (10). A major limitation of this study was the absence of a comparative native low altitude population. For practical reasons this was not possible, but by studying the LA Delhi population in the same detailed way as at HA, we were able to eliminate many recognised factors, leaving ancestry traits as a plausible mediator of the birthweight protection afforded to this population.

Maternal UtA diameter in the second trimester was strongly associated with birthweight in HA subjects. Women who went on to have AGA babies had larger UtA diameters at 18-24 weeks than women who went on to have a pregnancy complicated by SGA. This finding is independent of fetal sex differences. Similar UtA adaptation was not identified in a group of pregnant women at low altitude. UtA diameters were smaller and no difference was identified between women who had a pregnancy with either an AGA or SGA fetus. This supports the suggestion that one of the mechanisms through which HA adaptation maybe presenting in pregnancy is in early modulation of UtA diameter, enhancing blood flow, and thereby oxygen, to the developing placenta and fetus. In a separate cohort of high-altitude pregnancies studied, UtA blood flow was markedly greater at both weeks 20 and 36 of pregnancy in Andean versus European women, with such differences shown to be due to greater UtA diameters, not flow velocity (16).

Animal studies of the mechanisms underpinning UtA vasodilation supports a role for the metabolic sensor, adenosine monophosphate activated protein kinase (*AMPK*). *AMPK* was found to be present in utero-placental tissue and influenced vasodilation in the presence of chronic hypoxia (25) and its activation implicated in myometrial artery vasodilation in appropriate for gestational age human pregnancies at altitude in Colorado (26). Furthermore, pharmacological activation of AMPK in a murine model has been shown to partially present hypoxia induced fetal growth restriction (27). Peroxisome proliferator-activated receptor gamma (PPAR γ) has also been postulated to protect against hypoxia associated growth restriction with lower levels of expression in human growth restricted pregnancies compared with controls (28). Recently, using a murine model of hypoxia induced growth restriction, it was shown that exposure to a PPAR γ agonist was able to reverse induced vasoconstriction of uterine arteries with increased sensitivity of response in the hypoxic versus normoxic dams ((27, 29) which mimics our human physiological findings.

Given that gene variants in PRKAA1 (a subunit of AMPK) and PPAR γ expression differences associate with both birth weight and maternal UtA diameter, physiological mechanisms through which they may be acting warrant further investigation as they offer the potential to overcome hypoxia related growth issues.

## Conclusion

In the Ladakhi population, HA adaptation is represented through enhanced birth weight protection seen by lower than expected rates of SGA. Adaptation in pregnancy may be mediated through increased UtA diameter with resultant improved oxygenation to the placenta and fetus.

## Figures and Tables

**Table 1:** Maternal characteristics of low and high-altitude populations comparing controls (pregnant women who had appropriately grown babies) and cases (pregnant women who had small for gestational age babies <10^th^ centile customised to fetal gender and gestational age): Hb haemoglobin concentration; BP blood pressure; UtA uterine artery. Differences reported by unpaired t test. Mean ^+/^^-^ SD unless stated n (%)

Maternal Variables		*HIGH ALTITUDE*			*LOW ALTITUDE*		
		AGA (n=272)	SGA (n=44)	p value (95% CI)	AGA (n=82)	SGA (n=19)	p value (95% CI)
**Age (years)**		29.2 (4.6)	27.6 (4.6)	**0.03 (0.16-3.11)**	27.2 (2.9)	26.5 (3.6)	0.386 (-2.17- 0.84)
**Height (cm)**		154.5 (5.7)	155.2 (5.9)	0.438 (-1.09-2.55)	155.5 (6.4)	153.2 (4.6)	0.185 (-5.25- 1.02)
**Pre pregnancy weight (kg)**		53.5 (8.5)	50.6 (6.9)	0.134 (-6.63- 0.89)	56.3 (11.3)	49.3 (8)	0.012 (1.62-12.54)
**Booking Weight (kg)**		57.6 (9.4)	54 (8.2)	0.006 (1.2-7.1)	57.8 (10.8)	50.8 (7.9)	0.010 (1.69-12.23)
**BMI (Kg/m2)**		24.1 (3.7)	22.3 (3.1)	**0.002 (0.87-3.01)**	23.4 (4.4)	21.1 (3.4)	0.03 (0.15-4.44)
**Ethnicity (n/%)**		10 Tibetan. 2 Nepali 260 (96) Ladakhi >5 generations	2 Nepali 42 (96) Ladakhi 5 generations		78 Indoaryan 2 Dravidian 1 IndoDravidian 1 IndoEuropean	17 Indoaryan. 1 Dravidian 1 IndoEuropean	
**Ancestors not resident Leh/ Delhi n (%)**		2 (0.7)	2 (4.5)		40 (77)	11 (58)	
**Parity n (%)**	*Primiparous*	99 (36.4)	21 (47.7)		41 (50)	9 (47.4)	
	*Multiparous*	173 (72.6)	23 (52.3)		41 (50)	10 (52.6)	
**Past medical history**		7 Hypothyroid	1 Hypothyroid		2 Hypothyroid	1 Hypothyroid,1 Hypertension	
**Dietary n (%)**	*Meat/ all*	262 (96.3)	42 (95.5)		34 (41.5)	7 (36.8)	
	*Vegetarian*	10 (3.7)	2 (4.5)		48 (58.5)	12 (63.2)	
**Smoker (n)**		0	0		0	1	
**Education obtained**	*Nil formal*	20 (7.4)	4 (9)		4 (4.9)	2 (10.5)	
**n (%)**	*7 years*	19 (7)	4 (9)		4 (4.9)	2 (10.5)	
	*8-12 years*	192 (70.6)	27 (61.5)		64 (78)	14 (73.6)	
	*College/ university*	41 (15)	9 (20.5)		8(10.5)	1 (5.4)	
**Occupation**	*Housework*	166 (61)	25(57)		(65)	(58)	
**n (%)**	*Student*	6 (2.4)	2 (4.8)		(17)	(11.9)	
	*Government*	61 (22.3)	10 (22.7)		0	0	
	*Professional*	10 (3.7)	2 (4.5)		(4.5)	(6)	
	*Private*	29 (10.6)	5 (11)		(3)	(18)	
**Household income (Rs/month)**		28252 (19023)	29316 (18354)	0.99 (-5849- 8827)	43372 (38286)	30210 (35850)	0.19 (-31482- 6304)
**Hb (g/dl)**		12.11 (1.62)	12.36 (1.67)	0.51(-0.72 - 0.39)	11.79 (1.05)	12.09 (0.84)	0.225 (-0.89- 0.21)
**Blood glucose (mg/dl)**		87.9 (17.8)	88.3 (15)	0.67 (-10.1 to 6.6)	85.4 (8.2)	85.2 (8.1)	0.899 (-4.51- 3.96)
**Oxygen saturation (finger) %**		92 (1.94)	92.1 (1.83)	0.786 (-3.06 to 2.31)	98(1.02)	97.9 (0.96)	0.860 (-0.55- 0.66)
**Systolic BP (mmHg)**		111.2 (14.7)	111.4 (11.8)	0.944 (-5.04 to 5.42)	111.1 (10.2)	105.6(5.1)	0.046 (0.09-10.95)
**Diastolic BP (mmHg)**		73 (9.7)	74.2 (11)	0.527 (2.43-4.74)	71.5 (7.5)	66.8 (5.9)	0.025 (0.608-8.89)
		**n=187**	**n=34**		**n=47**	**n=14**	
**UtA diameter (mm)**		**56.5 (10.7)**	**51.5 (9.3)**	**0.009 (1.28-8.88)**	47.16 (4.9)	47.23 (4)	0.965 (-0.28- 0.29)

**Table 2:** Effect of pregnancy at high altitude on Ladakhi birth size and characteristics unless stated non significance between high altitude and low altitude groups. Difference between SGA (small gestational age) and AGA (appropriate for gestational age) babies within cohorts NS- non significant

ALTITUDE	HIGH	LOW	P value	(95% CI)
	n=316	n=101		
**Gender**
Male n/ %	144 (45.6%)	46 (45.5%)	NS	
Female n/ %	172 (54.4%)	55 (55.5%)	NS	

**Birth Weight (kg)**
Male	3.21(0.45)	2.95 (4.26)		
Female	3.10(0.46)	2.85 (3.75)		
**All**	3.15(0.44)	2.89 (0.4)	0.0001	0.178-0.376
*Tibetan (n=10)*	3.62 kg			
*Ladakhi (n=302)*	3.14 kg			
*Nepali (n=4)*	2.72 kg			
SGA (<10th centile)	2.52(0.32)	2.43 (0.22)		
AGA	3.25(0.39)	3.00 (0.36)	0.0001	0.157-0.034
				
**Birth Length(cm)**				
**All**	49.9 (2.6)	48.1 (1.7)	0.0001	1.111-2.343
SGA	47.5 (2.4)	47.1 (1.44)		
AGA	50.3 (0.15)	48.3 (0.19)		

**Birth weight/length**
<10th centile	53 (16.7%)	21 (19.8%)		

**Ponderal index (kg/m^3^)**
All	2.55 (0.35)	2.6 (0.27)	NS	

**APGARS 1 min**
All	8.26 (1.17)	8.47 (1.45)	NS	
SGA	8 (1.26)	7.79 (2.51)	NS	
AGA	8.3 (1.15)	8.62 (1.05)	NS	

**Finger O_2_ Sats (%)**
All	88.7 (8.32)	92.5(13.2)	0.005	-5.95 to -1.07
SGA	88.7 (7.18)	89.4 (14)	NS	
AGA	88.7 (8.49)	93.1 (13)	NS	

**Table 3:** Regression model evaluating factors associated with SGA outcome in high altitude population; column to the left reflects univariable analysis with variables of significance run in final multivariable model and values reported in righthand column OR odds ratio; Std Err standard error; CI confidence interval.

Variables	Univariable *OR*	*Std Err*	*P value*	*95% CI*	Final*OR*	*Std Err*	*P value*	*95% CI*
								
Ethnicity	0.634	0.26	0.283					
**Maternal Age**	1.08	0.040	**0.032**	1.01-1.16	1.07	0.047	0.125	
**Maternal weight (kg)**	1.06	0.228	0.012	1.01-1.1				
**Maternal BMI (kg/m^2^)**	1.14	0.058	**0.008**	1.03-1.26	1.10	0.058	0.085	
Parity	1.65	0.539	0.127					
Maternal Education	1.05	0.23	0.838					
Maternal Occupation	1.03	0.107	0.84					
**UtA diameter (mm)**	1.06	0.013	**0.0001**	1.03-1.08	1.06	0.013	**0.0001**	1.03-1.08
_cons					0.003	0.006	0.02	

## Impact

- Reduced fetal oxygen delivery seen in native lowlanders who gestate at high altitude causes fetal growth restriction- an effect thought to be mitigated in native high altitude [HA] populations
- We found that greater maternal body mass and uterine artery diameter were associated with increased offspring birth weight in a (Ladakh) HA population.
- This supports a role for them as physiological mediators of adaptation and provides insights into potential mechanisms that may treat hypoxia related growth issues.
